# Impact of retraining and data partitions on the generalizability of a deep learning model in the task of COVID-19 classification on chest radiographs

**DOI:** 10.1117/1.JMI.11.6.064503

**Published:** 2024-12-26

**Authors:** Mena Shenouda, Heather M. Whitney, Maryellen L. Giger, Samuel G. Armato

**Affiliations:** The University of Chicago, Committee on Medical Physics, Department of Radiology, Chicago, Illinois, United States

**Keywords:** COVID-19, deep learning, data partitioning, model generalizability, chest radiography

## Abstract

**Purpose:**

This study aimed to investigate the impact of different model retraining schemes and data partitioning on model performance in the task of COVID-19 classification on standard chest radiographs (CXRs), in the context of model generalizability.

**Approach:**

Two datasets from the same institution were used: Set A (9860 patients, collected from 02/20/2020 to 02/03/2021) and Set B (5893 patients, collected from 03/15/2020 to 01/01/2022). An original deep learning (DL) model trained and tested in the task of COVID-19 classification using the initial partition of Set A achieved an area under the curve (AUC) value of 0.76, whereas Set B yielded a significantly lower value of 0.67. To explore this discrepancy, four separate strategies were undertaken on the original model: (1) retrain using Set B, (2) fine-tune using Set B, (3) L2 regularization, and (4) repartition of the training set from Set A 200 times and report AUC values.

**Results:**

The model achieved the following AUC values (95% confidence interval) for the four methods: (1) 0.61 [0.56, 0.66]; (2) 0.70 [0.66, 0.73], both on Set B; (3) 0.76 [0.72, 0.79] on the initial test partition of Set A and 0.68 [0.66, 0.70] on Set B; and (4) 0.71±0.013 on repartitions of Set A. The lowest AUC value (0.66 [0.62, 0.69]) of the Set A repartitions was no longer significantly different from the initial 0.67 achieved on Set B.

**Conclusions:**

Different data repartitions of the same dataset used to train a DL model demonstrated significantly different performance values that helped explain the discrepancy between Set A and Set B and further demonstrated the limitations of model generalizability.

## 1 Introduction

In the early stages of the coronavirus disease 2019 (COVID-19) pandemic, deep learning (DL) algorithms emerged as potential tools for rapid diagnosis of the virus based on the chest radiographs (CXRs) of patients. As the deployment of these algorithms progressed, however, it became evident that their performance was not always consistent, and challenges arose in ensuring their reliability in clinical settings; in other words, the models were not generalizable. For example, some models were trained on CXRs of pediatric patients but were then applied to an adult population, which resulted in models predicting whether the patient was a child, not COVID-19 status.^1^ Similarly, a model trained on images of patients lying down and standing up was able to identify the status of patient positions, instead of disease status, with the intuitive notion that patients lying down were more likely to be ill.^2^ Further, most models struggled with robustness and generalizability, as there was poor truth labeling that sometimes relied on subjective assessments by physicians rather than more objective metrics such as reverse transcription polymerase chain reaction (RT-PCR) tests.^1^ Data collection was also a hindrance, as some available public datasets amalgamated data from various sources that may have included duplicate images, resulting in some CXRs being used in both the training and test sets, which yielded overly optimistic results.^1^^,^^2^ In all, a majority of models assessed early in the pandemic were not ready for clinical deployment, as there were inherent biases present.^1^^,^^3^

There were early efforts to combat data biases and lack of model generalizability. For example, to structure data curation, the Medical Imaging and Data Resource Center (MIDRC) was created with the aim “to foster machine learning innovation through data sharing for rapid and flexible collection, analysis, and dissemination of imaging and associated clinical data by providing researchers with unparalleled resources in the fight against COVID-19.”^4^ Further, MIDRC conducted a grand challenge to assess performance and generalizability of DL models in the task of distinguishing between COVID-19 positive/negative CXRs.^5^ Thus, the present study was motivated by continued scientific interest in model generalizability.^6^^–^^10^

Previous studies on model generalizability have quantified the impact of model deployment on out-of-distribution data in medical and non-medical environments.^9^^,^^10^ For example, Yang et al.^6^ studied models trained on electronic health record data across multiple clinical sites and concluded that models customized to a clinical site improved performance. However, McDermott et al.^7^ found that machine learning applied to healthcare tasks had poor reproducibility metrics (e.g., code and dataset accessibility). Further, Maleki et al.^8^ explored three common methodological pitfalls that reduce model generalizability. None of these studies, however, compared model performance and generalizability on imaging data within the same institution, nor provide an explanation for the appreciable decrease in model performance observed in the current work. The potential for bias even across datasets from the same institution is often overlooked.

A DenseNet-121 DL model previously published by Hu et al.^11^ (the “original model”) obtained an area under the receiver operating characteristic curve (ROC AUC) value of 0.76 in the task of COVID-19 classification; the same model achieved a significantly lower AUC value of 0.67 when applied to an independent test set from the same institution.^12^ This study, therefore, aimed to provide an interpretation for the outputs of the DL model in question, addressing the discrepancies between these two datasets by examining data partitioning, model architecture, and training, in an effort to understand the lack of model generalizability.

## 2 Methods

### 2.1 Datasets

#### 2.1.1 Set A

Set A included 9860 patients retrospectively collected from the University of Chicago Medicine under a Health Insurance Portability and Accountability Act (HIPAA)-compliant, Institutional Review Board (IRB)-approved protocol. The dataset was initially partitioned into 64% for training, 16% for validation, and 20% for testing using stratified sampling to maintain a consistent COVID-19 prevalence of 15.5% across the subsets. This training and validation set will be termed Set $A_{\mathrm{tr}}$, and the test set will be termed Set $A_{\mathrm{te}}$. Only the first CXR image acquired within 2 days of a patient’s initial RT-PCR test for the SARS-CoV-2 virus was used. CXRs were acquired between January 30, 2020, and February 3, 2021, using standard images from stationary dual-energy subtraction radiography units and portable radiography units. For further details on this dataset, refer to Hu et al.^11^

#### 2.1.2 Set B

CXR exams collected from 5893 patients constituted Set B and had been acquired between March 15, 2020, and January 1, 2022, under the same HIPAA-compliant, IRB-approved protocol. Within this cohort, 731 patients (12.4%) had tested positive, whereas 5162 patients (87.6%) had tested negative for the SARS-CoV-2 virus, as determined by RT-PCR tests. Patient images from both Set A (the initial set used to develop and evaluate the published model) and Set B (the newer set used to evaluate the published model) were obtained from the same institution and underwent identical image preprocessing. Although there was an overlap in image acquisition dates between Sets A and B due to the inherent patient curation process at the University of Chicago Medicine, there was no overlap in patients, and the two datasets were completely independent, as determined by the patient medical record numbers. The curation process for Set B paralleled that of Set A to mitigate the impact of potential confounding variables. For further details on this dataset, refer to Shenouda et al.^12^ Table 1 provides an overview of the two datasets.

**Table 1:** Number of patients and COVID prevalence for Set A and Set B.

	Number of patients	COVID prevalence (%)
Set $A_{\mathrm{tr}}$	7888	15.4
Set $A_{\mathrm{te}}$	1972	15.5
Set B	5893	12.4

### 2.2 Image Preprocessing

Digital Imaging and Communications in Medicine (DICOM) images of the CXR exams were normalized on the range [0, 255] and stored as Portable Network Graphics (PNG) images. Subsequently, an open-source U-Net-based model^13^ was used to segment the smallest rectangular region containing the lungs on the PNG images from both Set A and Set B. The segmentation model weights were computed using a pre-pandemic public CXR dataset^14^ and fine-tuned on another dataset featuring COVID-19 radiographs.^11^^,^^15^ Cropping was performed as it was shown to be effective for the original model^11^ and to maintain a consistent methodology.

### 2.3 Model Training Scheme

The current study is based on a model described by Hu et al.,^11^ which used a single, distinct partition of Set A: Set $A_{\mathrm{tr}}$ for the training and validation sets and Set $A_{\mathrm{te}}$ for the test set. The model employed a DenseNet-121 architecture,^16^ chosen for its previous success in diagnosing pneumonia and other pathologies on CXRs.^17^^,^^18^ In addition, it adopted a curriculum (transfer) learning approach,^19^ increasing the focus of the classification task towards COVID-19 in the final phase. The curriculum comprised three phases: (1) fine-tune the model pre-trained on ImageNet on the National Institutes of Health (NIH) ChestX-ray14 dataset,^20^^,^^21^ (2) refine on images from a pneumonia detection challenge,^22^ and (3) further fine-tune using the initial partition of Set A split into Set $A_{\mathrm{tr}}$ and Set $A_{\mathrm{te}}$.^11^

### 2.4 Analyses and Comparisons

**Fig. 1:**
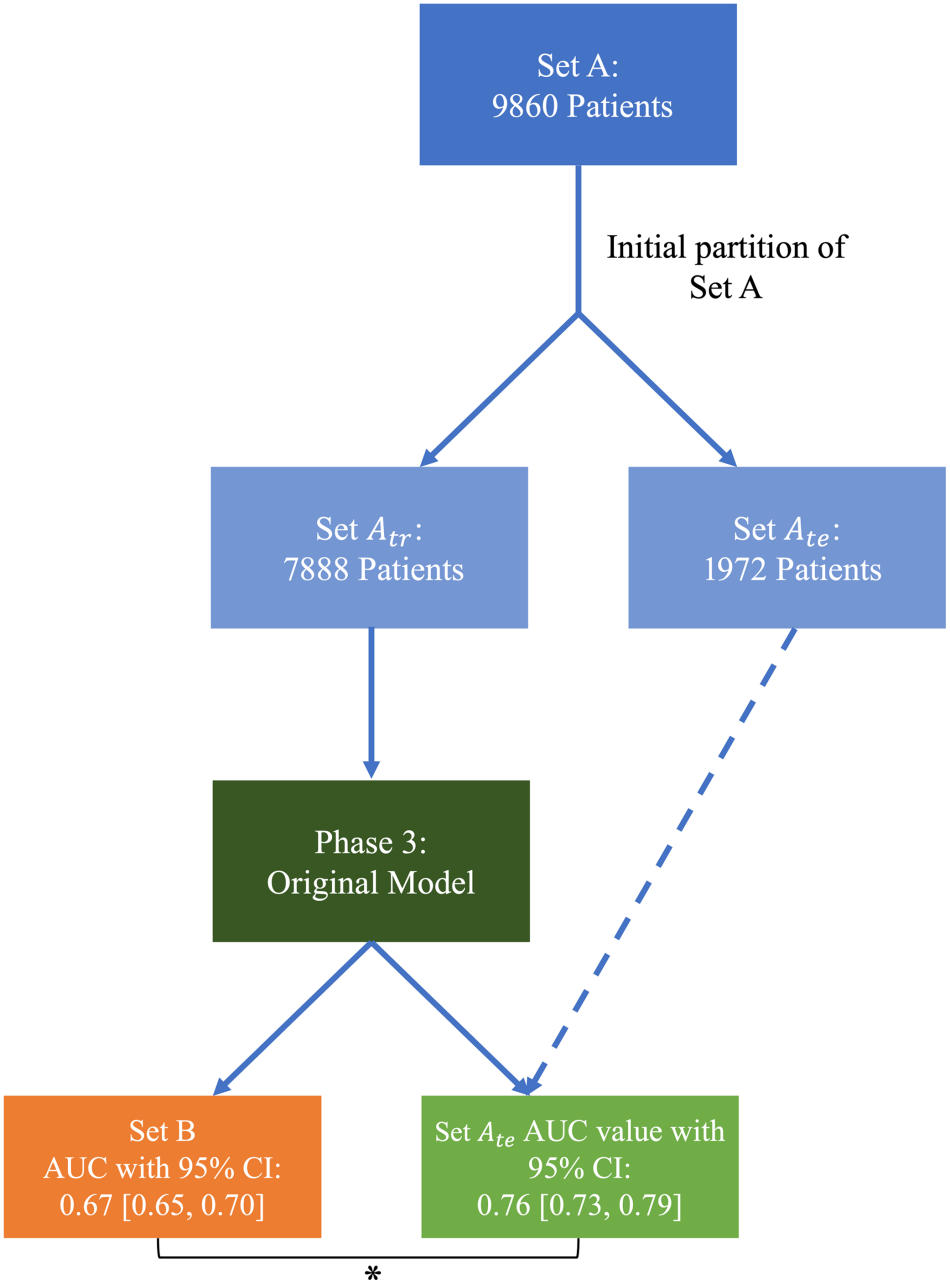
Comparisons performed between the initial partition of Set A and Set B. The AUC value refers to the test set of Set A, Set $A_{\mathrm{te}}$. The asterisk denotes the statistically significant difference between Set $A_{\mathrm{te}}$ and Set B. The green and orange boxes indicate results on Set $A_{\mathrm{te}}$ and Set B, respectively.

Using Set $A_{\mathrm{tr}}$ to train and validate, the original model yielded an AUC value of 0.76 [0.73, 0.79] (2000 bootstrapped samples to construct the 95% confidence intervals) on Set $A_{\mathrm{te}}$ in the task of distinguishing COVID+/- patients from their cropped standard CXRs. Using the same pre-trained model, Set B yielded an AUC value of 0.67 [0.65, 0.70] (also calculated from 2000 bootstrapped samples), which was significantly lower than the results of Set A (p<0.001) as determined by the DeLong test comparing the uncorrelated ROC curves (Fig. 1).^23^ To investigate the decrease in model performance from Set A to Set B, the present study investigated different model retraining strategies, an ablation technique, data partitioning (stratified sampling was used to maintain a consistent COVID-19 prevalence across all partitions), and model deployment on a grand challenge to assess model performance.

The first investigation, Experiment I, used Set B to retrain the model to calculate new phase 3 weights by employing the same split ratios as Set A: Set B was split into 64% training (3771 patients), 16% validation (943 patients), and 20% testing (1179 patients), using the cropped standard CXRs and maintaining the COVID prevalence at 12.4% across partitions. The combination of the Set B training and validation partitions will be termed Set $B_{\mathrm{tr},I}$, and the Set B test set will be termed Set $B_{\mathrm{te},I}$.

The second investigation, Experiment II, independently fine-tuned the model after the original phase 3 was conducted. Specifically, Set B was used to fine-tune the model after the original phase 3 weights by splitting the set into 40% training (2356), 10% validation (590 patients), and 50% testing (2947 patients). The combination of the Set B training and validation partitions will be termed Set $B_{\mathrm{tr},II}$, and the Set B test set will be termed Set $B_{\mathrm{te},II}$.

An ablation study, Experiment III, was also performed by altering the architecture of the original model for phase 3. Specifically, an L2 regularizer (with an L2 regularization penalty of 0.0005)^24^ was added to help mitigate overfitting, constraining the complexity of the model by minimizing the values the learned weights can take during phase 3. This was performed using the initial partition of Set A (Set $A_{\mathrm{tr}}$ and Set $A_{\mathrm{te}}$).

For Experiment IV, the phase 3 weights were recalculated for each of the 200 repartitions (determined empirically to calculate the two-sided 95% confidence interval) of Set $A_{\mathrm{tr}}$, and each of the resulting 200 models was evaluated on Set $A_{\mathrm{te}}$ and Set B to quantify the impact of data partitioning on performance. Specifically, the training and validation sets that comprise Set $A_{\mathrm{tr}}$ were separately resampled with replacement 200 times. These 200 partitions will be termed Set $A_{\mathrm{tr},IV}$ (i.e., 200 instantiations of Set $A_{\mathrm{tr},IV}$ were generated).

**Fig. 2:**
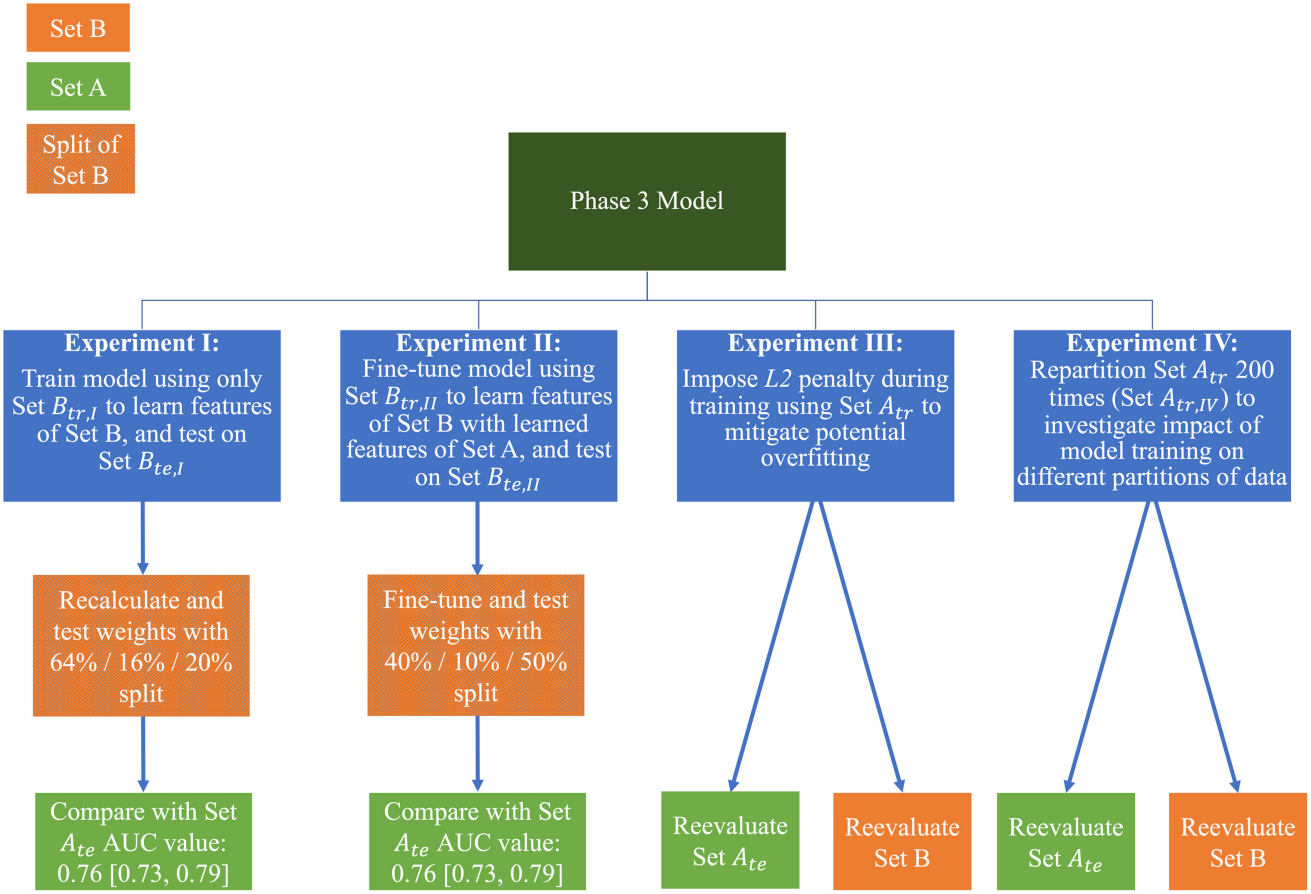
Summary of the four experiments conducted in this study.

Comparisons of model performance between Sets A and B were performed for standard CXRs when considering the four experiments: (1) recalculating the phase 3 weights using Set $B_{\mathrm{tr},I}$, (2) fine-tuning the phase 3 weights using Set $B_{\mathrm{tr},II}$, (3) implementing the L2 regularizer on the original model and retraining the phase 3 weights, and (4) repartitioning Set $A_{\mathrm{tr}}$ 200 times and recalculating the phase 3 weights, thereby evaluating whether the initial Set A results on Set $A_{te}$ were due to an initial chance favorable partitioning. Table 2 and Fig. 2 summarize the methods and comparisons performed.

**Table 2:** Summary of the datasets used and comparisons performed.

Experiment	Strategy or application	Training set	Comparison
I	Recalculating phase 3 weights	Set $B_{\mathrm{tr},I}$ (N=4714)	Set $A_{\mathrm{te}}$ (N=1972) and Set $B_{\mathrm{te},I}$ (N=1179)
II	Fine-tuning phase 3 weights	Set $B_{\mathrm{tr},II}$ (N=2946)	Set $A_{\mathrm{te}}$ (N=1972) and Set $B_{\mathrm{te},II}$ (N=2947)
III	L2 regularization applied during phase 3	Set $A_{\mathrm{tr}}$ (N=7888)	Set $A_{\mathrm{te}}$ (N=1972) and Set B (N=5893)
IV	200 repartitions and recalculating phase 3 weights	Set $A_{\mathrm{tr},IV}$ (N=7888)	Set $A_{\mathrm{te}}$ (N=1972) and Set B (N=5893)

## 3 Results

### 3.1 Experiment I: Recalculating Phase 3 Weights

After splitting Set B into a 64% training set, 16% validation set, and 20% test set while maintaining the COVID prevalence, the phase 3 weights were recalculated on Set $B_{\mathrm{tr},I}$ and a new AUC value of 0.61 [0.56, 0.66] was obtained on Set $B_{\mathrm{te},I}$ in the task of distinguishing COVID+/- when evaluating the cropped standard CXRs. This value is a significant decrease from 0.67 [0.65, 0.70] (p=0.029), which was obtained when applying the original model to the entirety of Set B. Further, this value was significantly lower than the initial Set $A_{\mathrm{te}}$ AUC value of 0.76 [0.73, 0.79] (p<0.001); though, Set $B_{\mathrm{tr},I}$ resulted in fewer images used for training (N=4714) when compared with Set $A_{\mathrm{tr}}$ (N=7888), which could explain the substantial decrease in the AUC values after recalculating the phase 3 weights using Set B.

### 3.2 Experiment II: Fine-tuning Phase 3 Weights

**Fig. 3:**
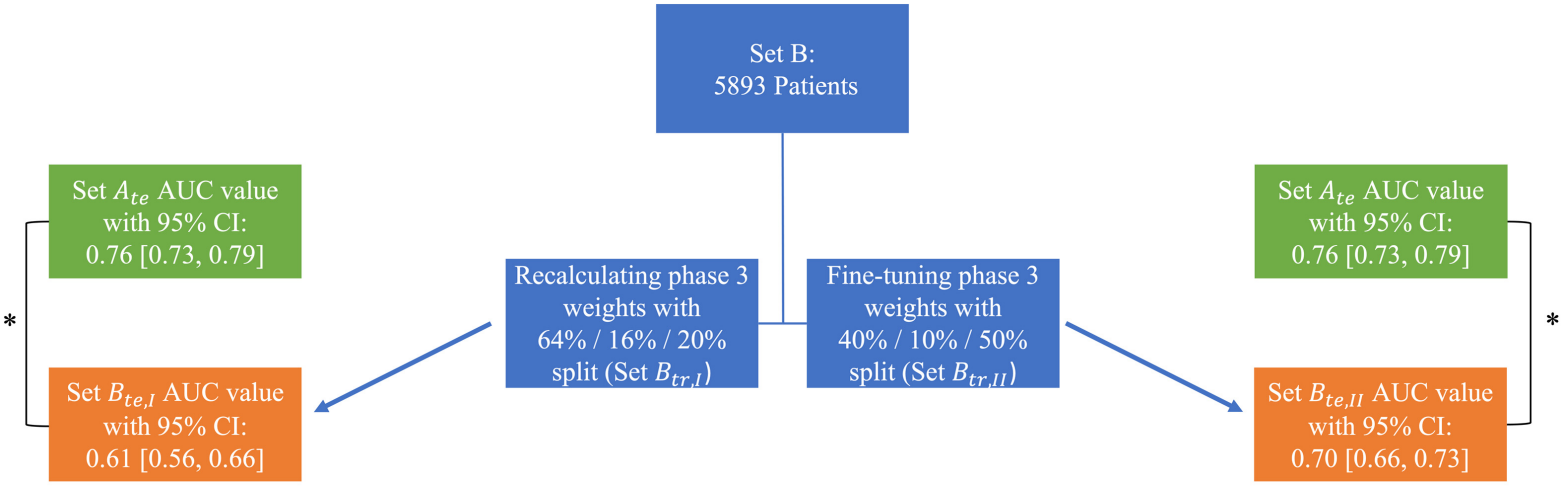
Summary of the results when recalculating (left) and fine-tuning (right) the phase 3 weights of the model. AUC values calculated in the task of distinguishing COVID+/- CXRs were significantly lower when comparing the partitioned Set B (Set $B_{\mathrm{te},I}$ and Set $B_{\mathrm{te},II}$) results with Set $A_{te}$, denoted by the asterisks. Green and orange boxes indicate results on Set A and Set B, respectively.

After fine-tuning the phase 3 weights by splitting the cropped standard CXR images of Set B into 40% training, 10% validation, and 50% testing while maintaining COVID prevalence, the AUC value calculated using Set $B_{\mathrm{te},II}$ slightly improved to 0.70 [0.66, 0.73] but was not significantly different from Set B without fine-tuning the phase 3 weights (AUC = 0.67, p=0.27). However, the 0.70 value was still significantly different from Set $A_{\mathrm{te}}$ (AUC = 0.76, p=0.007). A summary of the previous two comparisons is presented in Fig. 3.

### 3.3 Experiment III: L2 Regularization

Regularization did not mitigate model overfitting as the AUC values obtained with the regularized model failed to achieve a significantly higher AUC value than the corresponding AUC values prior to regularization for both Set $A_{\mathrm{te}}$ (0.76 [0.72, 0.79]) and Set B (0.68 [0.66, 0.70]).

### 3.4 Experiment IV: Recalculating Phase 3 Weights after Repartitioning

**Fig. 4:**
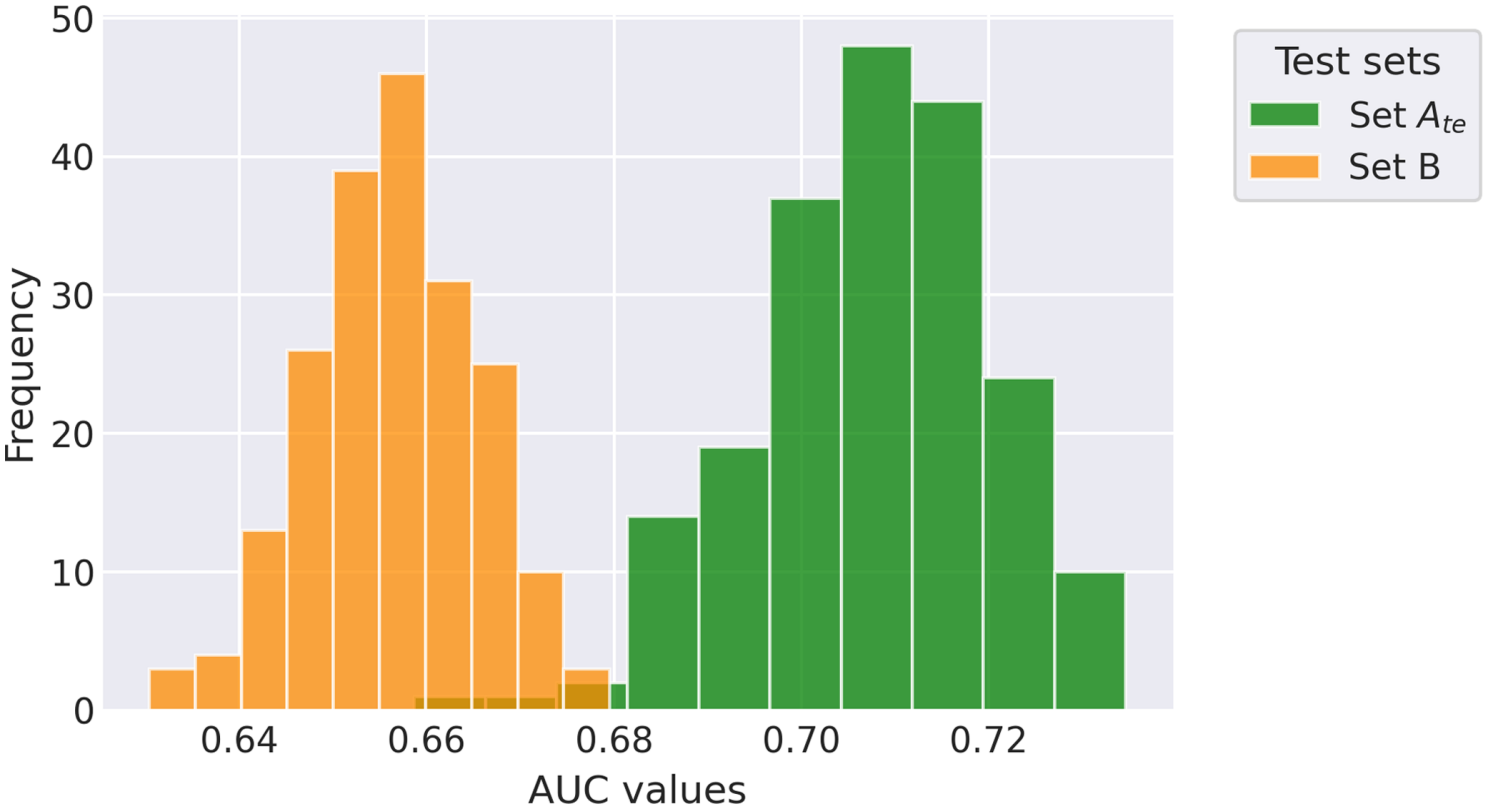
Distributions of the AUC values obtained when repartitioning Set $A_{\mathrm{tr},IV}$ 200 times and evaluating it on the test set of Set A, Set $A_{\mathrm{te}}$, and the entirety of Set B.

Retraining the model with the Set $A_{\mathrm{tr},IV}$ repartitions using the cropped standard CXR images resulted in an average AUC value of 0.71±0.013 on Set $A_{\mathrm{te}}$ and an average AUC value of 0.66±0.009 on Set B. There was a Gaussian-like distribution of AUC values for Set B (skew of –0.14) but a slight left-tailed distribution (skew of –0.46) for Set $A_{\mathrm{te}}$, as shown in Fig. 4. There was also a significantly larger variance of AUC values for Set $A_{\mathrm{te}}$ than for Set B (*F*-test, p<0.01), demonstrating the larger impact different training partitions had on Set $A_{\mathrm{te}}$ than Set B.

The lowest AUC value achieved on Set $A_{\mathrm{te}}$ during the 200 partitions was 0.66 [0.62, 0.69]. Interestingly, the initial AUC value of Set B (0.67 [0.65, 0.70]) was no longer significantly less than the AUC value obtained with this repartition of Set $A_{\mathrm{tr},IV}$ (p=0.46). Further, this lowest AUC value was significantly less than the initial Set $A_{\mathrm{te}}$ AUC value of 0.76 [0.73, 0.79] (p<0.001). The highest value achieved on Set $A_{\mathrm{te}}$ during the repartitions was 0.73 [0.70, 0.76], lower than the initial AUC value of 0.76, but this difference just failed to achieve statistical significance (p=0.069).

The lowest AUC value achieved on Set $A_{\mathrm{te}}$ from the aforementioned Set $A_{\mathrm{tr},IV}$ repartition (0.66 [0.62, 0.69]) was compared with its corresponding Set B AUC value (0.64 [0.62, 0.66]) on the exact same repartition and failed to achieve a significant difference (p=0.43). However, the highest AUC value achieved on Set $A_{\mathrm{te}}$ from the repartitions (0.73 [0.70, 0.76]) was significantly different from its corresponding Set B AUC value (0.65 [0.63, 0.68]) (p<0.001).

**Fig. 5:**
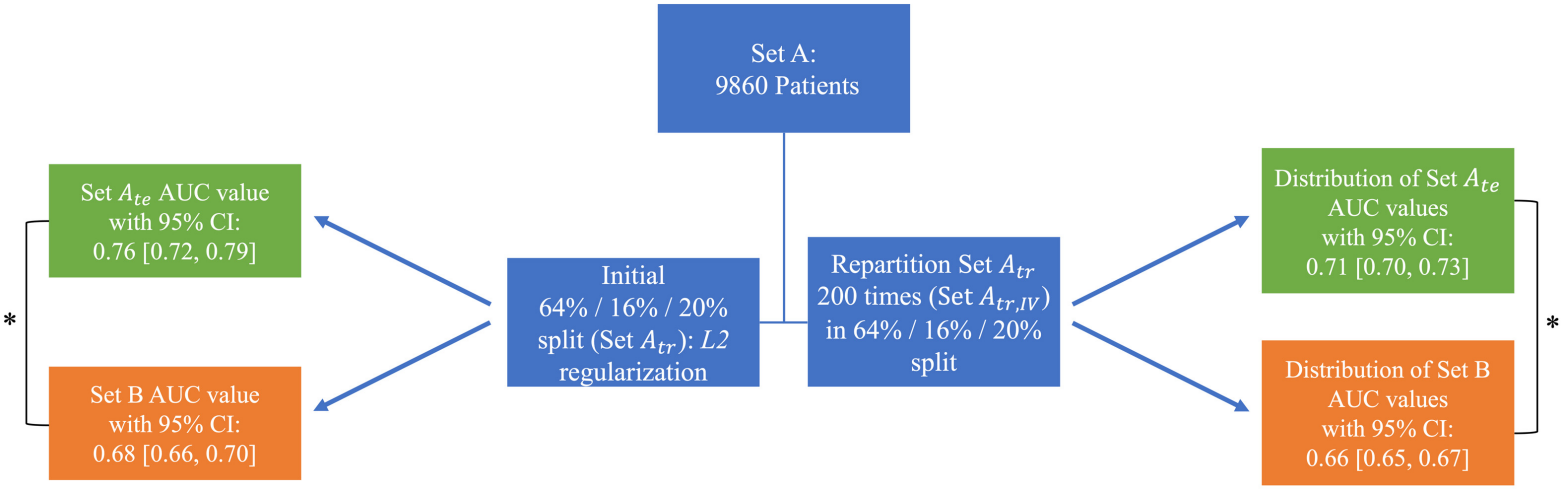
Summary of the results when implementing L2 regularization (left) and repartitioning Set $A_{\mathrm{tr}}$ 200 times (right). The AUC value of Set B was significantly lower than Set $A_{\mathrm{te}}$ for the L2 regularization, denoted by the asterisk. The distributions of the Set $A_{\mathrm{te}}$ AUC values and Set B AUC values obtained using the repartitioned Set $A_{\mathrm{tr},IV}$ achieved a significant difference, denoted by the asterisk. Green and orange boxes indicate results on Set A and Set B, respectively.

Distributions of AUC values resulting from the 200 partitions are displayed in Fig. 4, and a summary of the previous two analyses is presented in Fig. 5. There was a significant difference between the distributions of AUC values of Set $A_{\mathrm{te}}$ and Set B (Wilcoxon rank-sum test, p<0.001). A summary of the results from all the experiments is presented in Table 3.

**Table 3:** Summary of the main strategies or applications and their corresponding AUC values.

Experiment	Strategy or application	AUC values
I	Recalculating phase 3 weights	Set $B_{\mathrm{te},I}$: 0.61 [0.56, 0.66]
II	Fine-tuning phase 3 weights	Set $B_{\mathrm{te},II}$: 0.70 [0.66, 0.73]
III	L2 regularization applied during phase 3	Set $A_{\mathrm{te}}$: 0.76 [0.72, 0.79]
		Set B: 0.68 [0.66, 0.70]
IV	200 repartitions and recalculating phase 3 weights	Mean of Set $A_{\mathrm{te}}$: 0.71 [0.70, 0.73]
		Mean of Set B: 0.66 [0.65, 0.67]

## 4 Discussion

The motivation for this study was to examine the potential reasons behind the significant decrease in model performance between the initially partitioned Set A and Set B, which were both acquired at the same institution. Prior work discussed in Shenouda et al.^12^ extensively investigated this discrepancy in the performance of the model between the test sets as it explored the impact of age and sex, immunization status, COVID severity, type of imaging equipment, and date matching of image acquisition. None of these investigations, however, was able to explain the drop in performance. Assessment of artificial intelligence (AI) model performance on datasets acquired from the same institution provides a unique perspective on model generalizability. Therefore, this current work examined the impact of data partitioning and model retraining on performance and model generalizability.

Recalculating the phase 3 weights using Set $B_{\mathrm{tr},I}$ in Experiment I failed to improve performance of the model, perhaps due to the smaller number of cases on which the model trained compared with Set $A_{\mathrm{tr}}$, i.e., the original model trained on 6310 patients from Set A, whereas the recalculated phase 3 weights were trained on 3771 patients from Set B. Fine-tuning in Experiment II was performed to incorporate images from Set B in the training scheme (Set $B_{\mathrm{tr},II}$) in an attempt to improve model generalizability. Fine-tuning slightly improved the AUC value from 0.67 on Set B to 0.70 on Set $B_{\mathrm{te},II}$ for the cropped standard CXRs, but that value remained significantly lower than that of the initial Set $A_{\mathrm{te}}$ AUC value (0.76). The architecture of the model itself was altered in Experiment III in an attempt to create a more generalizable model. Specifically, L2 regularization was implemented to control for overfitting,^25^ although the regularization had a negligible impact on the performance of the model.

AUC values for Set $A_{\mathrm{te}}$ had a larger span (range: 0.66 to 0.73) than those of Set B (range: 0.63 to 0.68) when repartitioning Set $A_{\mathrm{tr}}$ 200 times during Experiment IV. Significant differences were achieved when comparing the highest AUC value calculated on Set $A_{\mathrm{te}}$ with its corresponding AUC value on Set B. Further, the highest Set $A_{\mathrm{te}}$ AUC value (0.73) failed to achieve a significant difference from the initial 0.76 AUC value, although it was lower. When analyzing the lowest AUC values, the difference between Set $A_{\mathrm{te}}$ and its corresponding Set B failed to achieve a significant difference. Differences between the lowest AUC value of Set $A_{\mathrm{te}}$ and the initial 0.67 AUC value of Set B also failed to achieve a significant difference. In other words, these values demonstrate that different partitions of the same dataset will yield significantly different results, returning variable performance. Therefore, although none of the patient demographics and clinical factors of the former study in Shenouda et al.^12^ could explain the decreased performance of the original model, the repartitioned results here indicate that a favorable, random partition may have been the reason for the discrepancy in performance. This work also emphasizes the “black box” nature of DL, as no discernible, real-world characteristic could explain the discrepancy of the model outputs. Instead, multiple repartitionings of the dataset demonstrated the large range of AUC values calculated, and consequently, the breadth of model performance and lack of generalizability. In addition, these results suggest that DL studies should report on model performance across multiple repartitions of the data, as that would provide a more reliable assessment of the model. Overall, the novelty of this work is its exhaustive and in-depth analysis investigating different training strategies to explain the decreased model performance when evaluating datasets that were acquired from the same institution.

Future work will explore further the creation of a generalizable model. This will include various regularization and augmentation methods. For example, test-time augmentation (TTA) could be employed by creating multiple augmented versions of the images in the test set. The model then makes predictions on each of these augmented versions, returning an ensemble of predictions, which can then be averaged. Specifically, test entropy minimization can be used to perform the TTA, as the minimization has been shown to reduce generalization error for image classification on corrupted ImageNet, ImageNet-C, and CIFAR-10/100 datasets.^26^ In addition, analyses in finding an optimal ratio of the data split into training, validation, and test sets will be conducted. Multiple studies^27^^–^^30^ have recommended a variety of splits, ranging from a 25% to a 50% split in creating the test set. Therefore, an optimal ratio will be explored to ensure the model is not overfit, which may result in improved generalizability. Lastly, an analysis of patient-based performance will be conducted. For instance, subset analyses (i.e., age or sex) can be performed on Set $A_{\mathrm{te}}$, and the classifier outputs, which varied with the different training repartitions, can be studied using a metric such as sureness introduced by Whitney et al.^31^ that evaluates the repeatability of the outputs. The metric can be used across different categories and across the two test sets, Set $A_{\mathrm{te}}$ and Set B.

## 5 Conclusion

This study examined a model trained to classify COVID-19 status based on patient CXRs and investigated the discrepancy in performance when the model was applied to two separate datasets acquired from the same institution, Set A and Set B. The model yielded significantly different AUC values between the initial test set Set $A_{\mathrm{te}}$ (0.76) and newer Set B (0.67). Methods and modifications of model architecture, model retraining, and model fine-tuning were all performed in an attempt to explain the lower AUC value. The exploration of data partitioning was able to provide an explanation for the decreased performance between the datasets, as it underscored the variability introduced by different partitions.

As DL algorithms become more widespread in healthcare tasks, it is imperative that AI scientists understand and interpret the outputs of these models to explain and mitigate potential inconsistencies in model performance. Overall, this work contributes to the methods of explainable AI, as it attempts to interpret the results of the DL algorithm used to classify COVID-19 status. These findings emphasize the need for continued research in improving model training, fine-tuning, and augmentation to address model generalizability before deployment in the clinic.

## Data Availability

A collection of data presented in this article is now publicly available at https://data.midrc.org/ The archived version of the code described in this manuscript can be freely accessed through GitHub.
